# Chronic circadian misalignment accelerates immune senescence and abbreviates lifespan in mice

**DOI:** 10.1038/s41598-020-59541-y

**Published:** 2020-02-13

**Authors:** Hitoshi Inokawa, Yasuhiro Umemura, Akihiro Shimba, Eiryo Kawakami, Nobuya Koike, Yoshiki Tsuchiya, Munehiro Ohashi, Yoichi Minami, Guangwei Cui, Takuma Asahi, Ryutaro Ono, Yuh Sasawaki, Eiichi Konishi, Seung-Hee Yoo, Zheng Chen, Satoshi Teramukai, Koichi Ikuta, Kazuhiro Yagita

**Affiliations:** 1 Department of Physiology and Systems Bioscience, Kyoto Prefectural University of Medicine, Kyoto, 602-8566 Japan; 2 Laboratory of Immune Regulation, Department of Virus Research, Institute for Frontier Life and Medical Sciences, Kyoto University, Kyoto, 606-8507 Japan; 3 Medical Sciences Innovation Hub Program, RIKEN Center for Integrative Medical Sciences, Yokohama, Kanagawa 230-0045 Japan; 4 Artificial Intelligence Medicine, Graduate School of Medicine, Chiba University, Chiba, 260-0856 Japan; 5 Department of Surgical Pathology, Kyoto Prefectural University of Medicine, Kyoto, 602-8566 Japan; 6 Department of Biochemistry and Molecular Biology, The University of Texas Health Science Center at Houston, 6431 Fannin St., Houston, TX 77030 USA; 7 Department of Biostatistics, Kyoto Prefectural University of Medicine, Kyoto, 602-8566 Japan; 8 Graduate School of Medicine, Kyoto University, Kyoto, 606-8501 Japan

**Keywords:** Ageing, Transcriptomics

## Abstract

Modern society characterized by a 24/7 lifestyle leads to misalignment between environmental cycles and endogenous circadian rhythms. Persisting circadian misalignment leads to deleterious effects on health and healthspan. However, the underlying mechanism remains not fully understood. Here, we subjected adult, wild-type mice to distinct chronic jet-lag paradigms, which showed that long-term circadian misalignment induced significant early mortality. Non-biased RNA sequencing analysis using liver and kidney showed marked activation of gene regulatory pathways associated with the immune system and immune disease in both organs. In accordance, we observed enhanced steatohepatitis with infiltration of inflammatory cells. The investigation of senescence-associated immune cell subsets from the spleens and mesenteric lymph nodes revealed an increase in PD-1^+^CD44^high^ CD4 T cells as well as CD95^+^GL7^+^ germinal center B cells, indicating that the long-term circadian misalignment exacerbates immune senescence and consequent chronic inflammation. Our results underscore immune homeostasis as a pivotal interventional target against clock-related disorders.

## Introduction

From the cellular to the organismal levels, circadian clocks regulate various essential biological processes to enable anticipation of and adaptation to the daily environmental changes from Earth rotation^1^. Modernization of our society is accompanied by a dramatic change in human lifestyle, with unprecedented increases in, for example, night shift work and nocturnal feeding/recreational activities^2^. Recent epidemiological studies have revealed shift workers as being at a higher risk of various diseases, such as mood disorders, metabolic syndrome, cardiovascular disease, and some types of cancers, suggesting that the misalignment between environmental cycles and endogenous circadian clocks exacerbates systemic pathological consequences^3–8^. However, the pathophysiological mechanisms underlying the deleterious effects of long-term circadian misalignment in health and healthspan remain unclear.

Recent studies have investigated the perturbation of circadian systems by environmental and/or genetic manipulation in animal models^9,10^. For example, Davidson *et al*. reported that an experimental model of environmental perturbation induced by the scheduled shifts of light–dark cycles —called chronic jet-lag (CJL)— for 8 weeks using aged mice (27–31 months old) showed the mortality rate to be higher in the phase advance condition (6-hour phase advance every 7 days) than in the phase delay (6-hour phase delay every 7 days) condition or control LD condition^9^. These studies principally investigated the acute or subacute (for up to a few months) effects of circadian misalignment; it thus remains uncertain how long-term perturbation of environmental light-dark cycle induces physiological transformation and pathological consequences.

In mammals, the suprachiasmatic nucleus (SCN) functions as the center of a circadian regulation system to coordinate the cell-autonomous clocks in peripheral organs or tissues throughout the body^11–14^. Light is one of the most important environmental elements entraining the internal circadian system of mammals to the external cycle^15,16^; the SCN receives the neural projection from retinal ganglion cells expressing the melanopsin photoreceptor, through which mammals are entrained by light–dark cycles^17–19^. Moreover, experimental models of environmental perturbation induced by CJL may reportedly result in the desynchronization of circadian rhythms within the SCN^20,21^. Therefore, the disruption of the SCN rhythm and the ensuing misalignment between the SCN and peripheral organs have been considered to be a trigger of circadian misalignment-associated health problems. However, studies investigating the systemic effects of long-term CJL in animal models are sparse; thus, the etiology of health problems by chronic circadian misalignment has not yet been fully uncovered.

In this study, we established a prolonged CJL paradigm in mice to interrogate the function and mechanism of long-term (~85 weeks) circadian misalignment in mice. We exposed mice to a non-adjustive light–dark shift condition (8-hour phase advance every 4 days; ADV) or an adjustive shift condition (8-hour phase delay every 7 days, DEL), with a control group under the normal light-dark condition. Interestingly, our results revealed significantly shorter lifespan in ADV mice, correlated with perturbed SCN rhythms. Our mechanistic studies highlighted a key mechanistic role of accelerated immune senescence for CJL-related mortality.

## Results

### Long-term circadian misalignment induces a decrease in survival rate in mice

Our recent preliminary pilot study surveying the effects of long-term circadian misalignment on mouse physiology has raised the possibility that the long-term circadian misalignment by an 8-hour advance every 4 days (ADV) condition tended to lead shorter lifespan in mice than DEL condition^22^. Therefore, we first performed a chronic, rigorous test to determine the role of CJL on the lifespan of adult wild-type mice. In this study, we exposed the mice to two distinct CJL conditions differing in the timing of light on/off—an 8-hour delay every 7 days (DEL) and an 8-hour advance every 4 days (ADV), in addition to the control light–dark condition with an 8:00 h–20:00 h light period (LD)^22^ (Fig. S1). We used these ADV and DEL condition to induce non-adjustive and adjustive phase shifts in the behavior. DEL-conditioned mice were re-synchronized to the shifted light–dark cycle within a few days after 8-hour phase delay (Fig. S1). On the contrary, ADV-conditioned mice were unable to resynchronize their activity to the shifted light–dark cycle and, therefore, suffered from continuous non-adjustive and/or severely disturbed behavioral rhythms.

**Figure 1:**
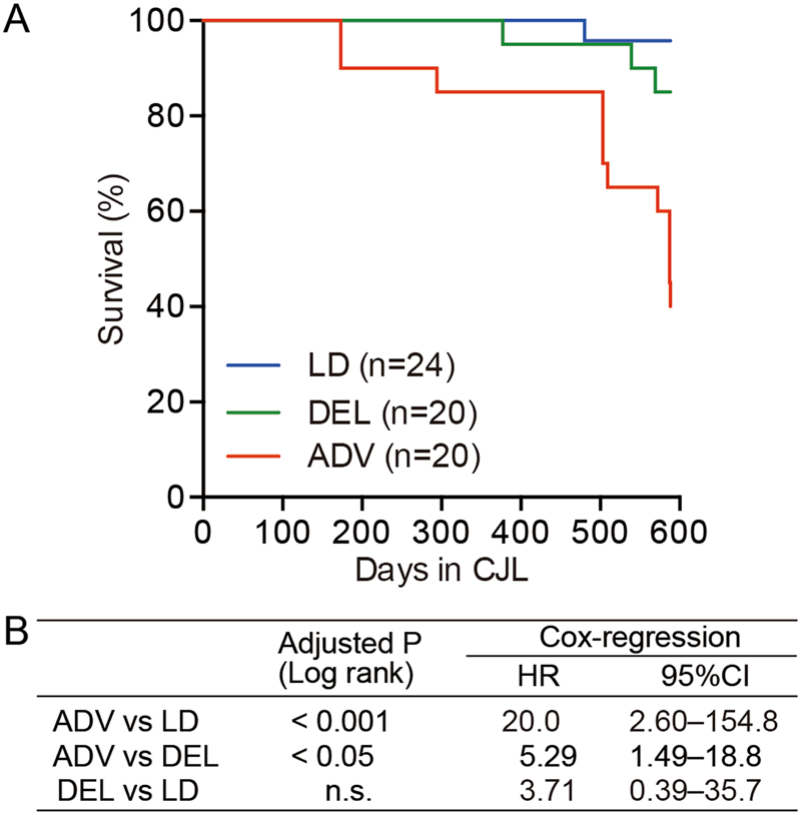
The long-term non-adjustive condition of light–dark cycles led to increased mortality. (**A**) Kaplan–Meier survival curves of mice kept under the three conditions. (**B**) The log-rank test was used to compare the Kaplan-Meier curves for the indicated conditions. The *P* values were adjusted by Bonferroni’s correction. The Cox proportional hazard regression model stratified by light conditions was used to obtain the hazard ratio (HR) and the associated 95% confidence interval (95% CI). n.s. = 0.6684.

We observed that the lifespan of the ADV-conditioned mice was shortened with statistical significance, comparing with that of both LD- and DEL-conditioned mice (Fig. 1A). Stratified Cox regression analysis revealed the mortality rate of ADV-conditioned mice to be 20.0 times higher than that of LD-conditioned mice (*P* < 0.001) and 5.29 times higher than that of DEL-conditioned mice (*P < *0.05) (Fig. 1B). Interestingly, however, the Kaplan-Meier survival curve showed no significant difference between DEL- and LD-conditioned mice (Fig. 1B). Although the specific mortal causes were unclear, these results established a specific circadian disruption (ADV) that abbreviates the lifespan of adult wild-type mice.

### The ADV condition induced intra-SCN circadian desynchronization

**Figure 2:**
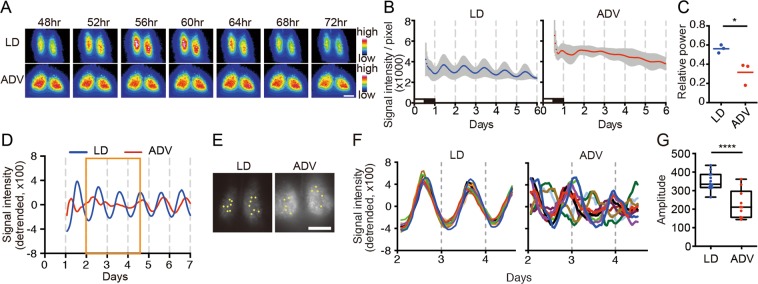
The long-term non-adjustive ADV-condition desynchronized SCN neurons. (**A**) Representative bioluminescence images of SCN slice culture from *Per2*^*Luc*^ mice under the indicated conditions for ~1 year. The animals were sampled at ZT12 on the second day after the shift. Hours indicate time after the last light onset. Scale = 200 µm. (**B**,**C**) Averaged bioluminescence traces from SCN slice cultures (mean ± SD, n = 3) and the FFT spectral analysis of the traces. Two-tailed Student’s *t*-test, *P < 0.05. Black and white bars show the animal’s previous LD conditions. (**D**) The detrended data of whole bioluminescence traces of the SCN slice culture from the indicated conditions. Orange boxes indicate the duration used for the single-cell level analysis. (**E**) ROIs for single-cell bioluminescence traces were represented in SCN images from each condition. LD, n = 14; ADV, n = 10. Scale = 250 µm. (**F**,**G**) The detrended traces at the single-cell level and the amplitude of the detrended traces. Two-tailed Student’s *t*-test, ****P < 0.0001.

To investigate the underlying basis for the severe consequences of ADV, we first examined the circadian oscillation in the central pacemaker SCN using *mPer2::Luciferase* knock-in mice (*Per2*^*Luc*^). We performed acute slice culture of the hypothalamus including the SCN obtained from *Per2*^*Luc*^ mice of long-term exposure to ADV and LD conditions, and analyzed the bioluminescence rhythms in both whole SCN and single-cell levels. Since it has been reported that the age of mice affects the synchronicity of intra-SCN neuronal rhythms^23,24^, we prepared similar aged mice to evaluate the SCN rhythm against the long-term circadian misalignment. For example, SCN of ADV-conditioned mice (110-, 90-, 65-week-old (wo)) were compared with SCN of LD mice (112-, 108-, 103-wo). Whole SCN-level bioluminescence imaging revealed severe impairment of the circadian oscillation in the SCN from all ADV-conditioned mice, whereas robust circadian bioluminescence oscillation persisted in the SCN of similar aged LD mice. (Fig. 2A–C). In contrast to the study involving single phase shift in rats^25^, we did not observe the desynchrony in the phase relationship between ventrolateral and dorsomedial regions of SCN in the ADV-conditioned mice (Fig. 2A), suggesting that the intra-SCN desynchronization here is due to the long-term shifting. Single-cell-level analysis revealed that each neuron exhibited apparent circadian oscillation—even in the ADV condition—but the phases of analyzed ADV cells were widely dispersed and the overall amplitudes were lower even in relatively younger (65-wo) mouse (Fig. 2D–G), suggesting that intra-SCN desynchronization in mice with long-term exposure to the non-adjustive ADV perturbation of light-dark condition. These findings showed that the long-term ADV-conditioned mice displayed circadian desynchronization within the SCN, strongly suggesting a causal relationship between circadian disruption and early mortality.

### Comprehensive analysis of the long-term circadian misalignment-conditioned peripheral organs

The failure of tuning the peripheral clocks via the autonomic nervous system and/or endocrine system, as a result of SCN desynchronization, has been shown to initiate various systemic physiological dysfunctions^2,26,27^. The liver is a central peripheral organ whose functions are regulated by the circadian system^26^. We therefore performed non-biased evaluation using polyA-selected RNA sequencing (RNA-seq) on liver tissues obtained from 85-week ADV-conditioned and LD-conditioned mice (aged 97 weeks) without apparent weakness or injury. Expression levels of the core clock genes are divergent, but comparable between LD- and ADV-conditioned mice (Fig. S2).

**Figure 3:**
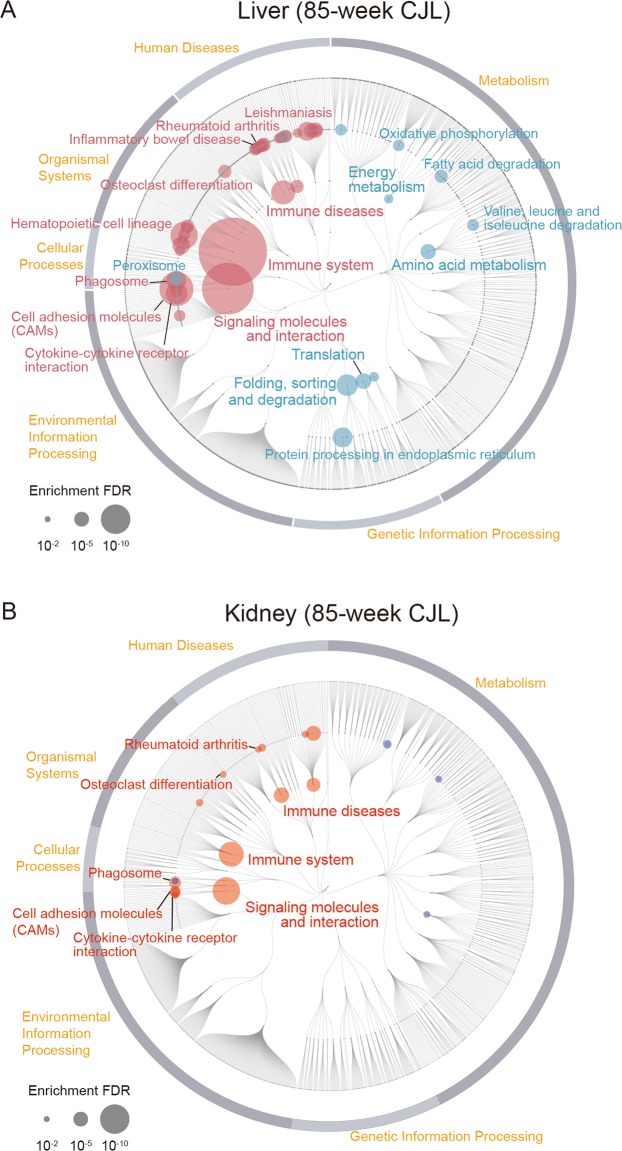
RNA-seq of gene expression in livers and kidneys from mice kept under LD- and non-adjustive ADV-conditions for 85 weeks. (**A**,**B**) Enrichment analysis based on KEGG functional hierarchy for gene expression in the ADV-conditioned liver (**A**) or kidney (**B**) relative to their expression in the LD-condition. Node size indicates the false-discovery rate (FDR) of the enrichment analysis. Red nodes indicate significantly upregulated pathways in the ADV condition, while blue ones indicate significantly downregulated pathways.

To interrogate cellular mechanisms, we performed KEGG pathway enrichment analysis between ADV and LD groups. Our analysis revealed a wide spectrum of alteration in functional pathways in the 85-week ADV-conditioned liver when compared with those of the LD-conditioned livers. Interestingly, however, in the ADV condition, immune diseases and inflammation-related pathways, such as rheumatoid arthritis and inflammatory bowel disease associated pathways, were markedly activated, while metabolic pathways of lipids and amino acids were extensively repressed (Fig. 3A).

We also performed KEGG pathway enrichment analysis of kidney gene expression between the groups (Fig. 3B). Interestingly, as in the liver, pathways associated with immune system and immune diseases in the ADV-conditioned kidney—unlike those in LD-conditioned kidneys—were also activated. These results provide evidence for a systemic enhancement of inflammatory response in 85-week ADV-conditioned mice.

**Figure 4:**
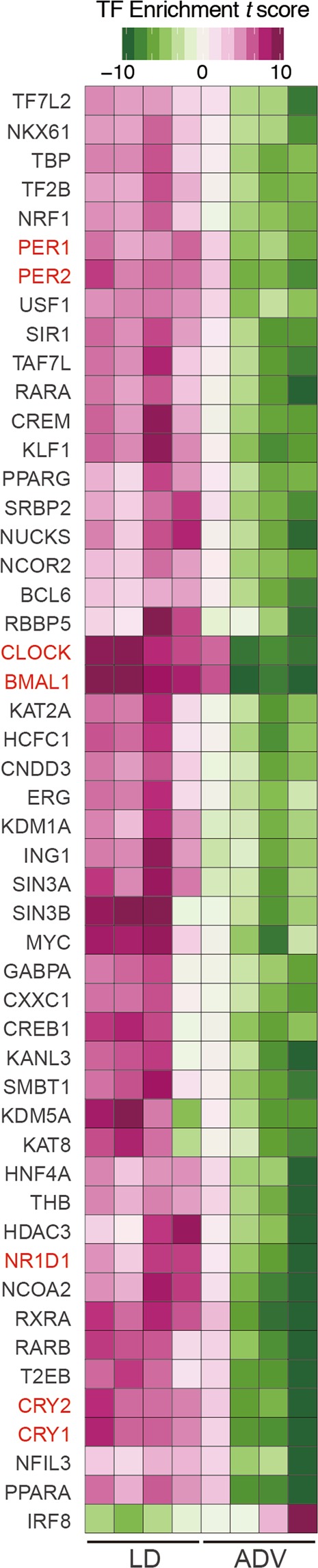
TF enrichment analysis of long-term non-adjustive ADV-conditioned livers for 85 weeks. Gene sets enrichment analysis for evaluating effects of TFs on their binding target genes. We used normalized expression values of genes to calculate the relative effects of TFs in each sample. Estimated effects of TFs are presented in heatmaps as enrichment *t*-score.

We further investigated the alteration of gene regulatory networks in the liver induced by the ADV condition with transcription factor (TF) enrichment analysis (Fig. 4). Gene expression modules regulated under core circadian transcriptional factors—including BMAL1, CLOCK, PER1, PER2, CRY1, CRY2, and NR1D1—were significantly suppressed in the ADV-conditioned liver compared with those in the LD condition, despite the similar expression levels of these core clock genes between LD and ADV mice (Fig. S2). Therefore, these results suggest the gene regulatory networks controlled by the circadian feedback loops are suppressed in the ADV-conditioned liver.

**Figure 5:**
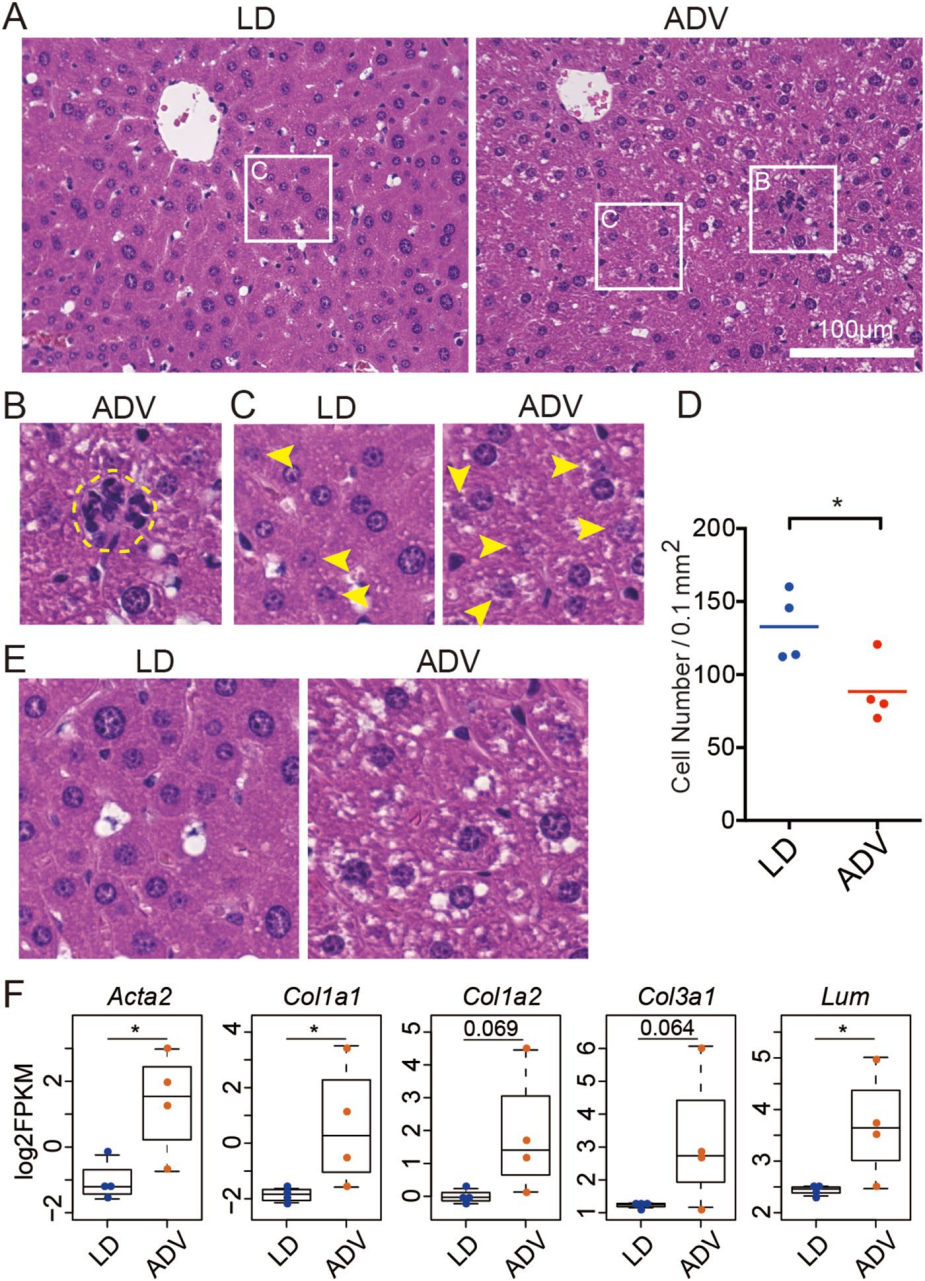
Histological analysis of livers from mice kept under long-term ADV conditions for 85 weeks. (**A**) H&E staining of the livers from long-term ADV-conditioned mice. Each squared portion is magnified in (**B**,**C**). (**B**) Representative images of hematopoietic cell infiltration in livers of mice kept under long-term non-adjustive ADV condition. (**C**) Arrow heads indicate the degeneration of liver parenchymal cells which contain fragmented nuclear materials. (**D**) Quantification of the number of normal cells per unit area under each condition (n = 4). One dot indicates the average of four different visual fields in each mouse liver. Two-tailed Student’s *t*-test, *P < 0.05. (**E**) Fat deposition was accelerated in the long-term non-adjustive ADV-conditioned liver. (**F**) Beeswarm box plots of gene expression levels related to the fibrosis-associated genes. Asterisks indicate DESeq2 significance (FDR <0.05).

We next examined the histology of these liver tissues (Fig. 5). Consistent with gene expression analysis above, clusters of infiltrating inflammatory cells were enhanced in the ADV mice, indicating low-grade chronic inflammation (Fig. 5A,B). Furthermore, a significant reduction in the number of liver parenchymal cells surrounding the central veins in the ADV-conditioned mice when compared with those of the LD-conditioned mice suggested that the degeneration of liver parenchymal cells was enhanced by long-term ADV exposure (Fig. 5C,D). In ADV-conditioned mice, fat deposition in the liver was also increased, implying hepatic steatosis (Fig. 5E). These results show that low-grade chronic inflammation in liver parenchymal cells is caused by exposure to the long-term non-adjustive CJL condition, supporting the results from comprehensive gene regulatory network analysis.

At the molecular level, the expression of fibrosis-associated genes—alpha-actin-2 (*Acta2*), also known as alpha-smooth muscle actin (α-SMA), collagens (*Col1a1*, *Col1a2*, and *Col3a1*) and lumican (*Lum)* was upregulated in the ADV-conditioned livers, as detected by the comprehensive gene expression analysis using RNA-seq (Fig. 5F). Consistent with the histological analysis, these results suggest that long-term non-adjustive CJL-conditioning promotes low-grade chronic inflammation with fibrosis similar to chronic steatohepatitis.

### Long-term Circadian Misalignment conditioning accelerates immune senescence

**Figure 6:**
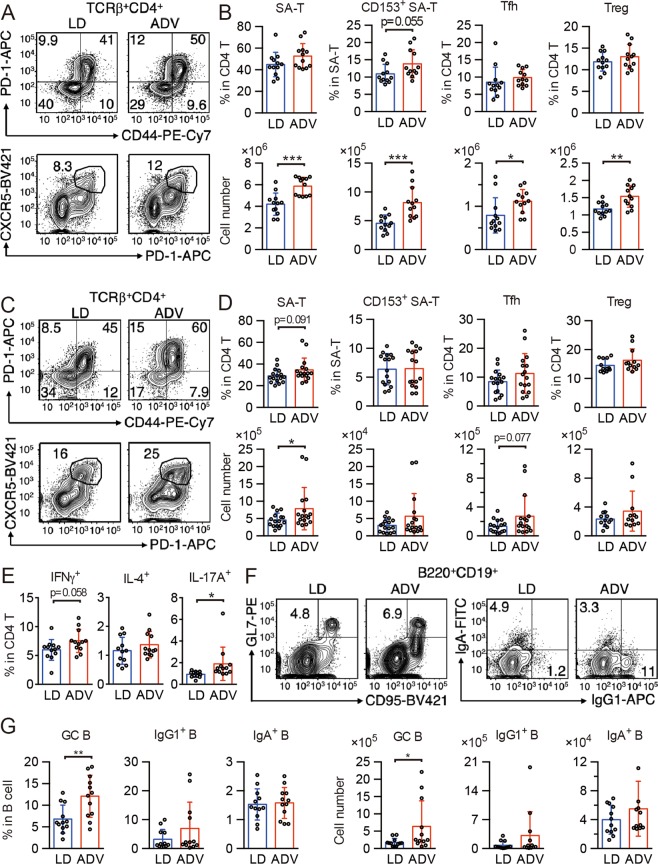
The long-term non-adjustive ADV condition accelerates generation of senescence-associated (SA) T cells and germinal center B cells. (**A**,**B**) Flow cytometry of senescence-associated T (SA-T) and follicular helper T (Tfh) cells in CD4^+^TCRβ^+^-gated spleen cells (**A**), and percentages and cell numbers of SA-T (CD4^+^TCRβ^+^CD44^+^PD-1^+^), CD153^+^ SA-T, Tfh (CD4^+^TCRβ^+^CXCR5^+^PD-1^+^), and regulatory T (Treg, CD4^+^TCRβ^+^CD25^+^) cells (B, n = 12) in spleens of LD- and ADV-conditioned mice. (**C**,**D**) Flow cytometry of SA-T and Tfh cells in CD4^+^TCRβ^+^-gated mLN cells (**C**) and percentages and cell numbers of SA-T, CD153^+^ SA-T, Tfh, and Treg (D, n = 12–16) in mLNs of LD- and ADV-conditioned mice. (**E**) Whole mLN cells from LD- and ADV-conditioned mice were stimulated with PMA and ionomycin for 3 hours. Percentages of IFN-γ–, IL-4–, and IL-17A–producing helper T cells in CD4 T cells were shown (n = 12). (**F**) Flow cytometry of germinal center B cell (GC-B) and IgG1^+^ and IgA^+^ class-switched B cells in mLNs of LD- and ADV-conditioned mice. (**G**) Cell numbers of GC-B (CD19^+^B220^+^CD95^+^GL7^+^), IgG1 B cells (CD19^+^B220^+^IgG1^+^), and IgA B cells (CD19^+^B220^+^IgA^+^) in mLN from LD- and ADV-conditioned mice (n = 12). Data are means ± SD. Two-tailed Student’s *t*-test, *P < 0.05, **P < 0.01, ***P < 0.001.

In addition to the activated pathways associating with immune system and diseases, our recent pilot study has also indicated that ADV mice with early death often exhibited severe inflammation at their humane endpoints^22^. These findings raised the possibility that the pathophysiology of chronic circadian misalignment induced by the long-term ADV condition may be associated with immune dysfunction. To determine whether the chronic inflammation we observed correlates with immune senescence^28^, we next analyzed senescence-associated (SA) immune cell subsets in the spleen and mesenteric lymph nodes (mLNs) from middle-aged mice (aged 77 weeks) exposed to CJL conditions for 65 weeks. As mice age, frequencies of PD-1^+^CD44^high^ CD4 T cells (SA-T cells)^29^, CD153^+^ SA-T cells^30^, follicular helper T cells (Tfh cells)^30^, and regulatory T cells (Treg cells)^31^ increase. Although lymphocyte development was unchanged (Fig. S3), SA-T, CD153^+^ SA-T, Tfh, and Treg cells increased in the spleen (Fig. 6A,B), and SA-T and Tfh cells increased in mLNs in ADV-conditioned mice (Fig. 6C,D), compared with those of LD-conditioned mice. Next, we cultured mLN cells to differentiate into type 1 helper T (Th1), Th2, and Th17 cells. Differentiation of IL-17A^+^ Th17 cells in ADV-conditioned mice was elevated, and that of IFNγ^+^ Th1 cells tended towards elevation (but this trend was not significant), whereas that of IL-4^+^ Th2 cells was unchanged (Fig. 6E). These results indicate that chronic circadian misalignment accelerates T cell senescence and enrichment of Th1 and Th17 cells.

SA-T and Tfh cells in aged mice stimulate B cells and induce germinal center (GC) formation and class switch^30,32^; we therefore examined GC B and class-switched B cells. In ADV-conditioned mice, the frequency and number of CD95^+^GL7^+^ GC B cells increased, and those of class-switched IgG1^+^ B cells showed a trend to increase (p = 0.19 or 0.18) when compared with those of LD-conditioned mice (Fig. 6F,G). These results provide evidence for the activation of Tfh cells and the acceleration of GC formation and class switch as a result of chronic circadian misalignment. SA-T cells produce high amounts of osteopontin, causing inflammation and autoantibody production^29,30,32^, and Th1 and Th17 cells exacerbate autoimmunity^33^. Our results suggest that by enhancing generation of Th1 and Th17 cells, long-term exposure to non-adjustive CJL condition aggravates inflammation and autoimmunity. Consistently, KEGG pathway enrichment analysis in the livers and kidneys of 85-week ADV-conditioned mice indicated the activation of gene regulatory pathways associated with autoimmune diseases such as rheumatoid arthritis (Fig. 3).

Since circadian rhythms regulate innate immunity^34^ and circadian disruption increases production of pro-inflammatory cytokines in myeloid cells after LPS stimulation^35^, we next analyzed myeloid cell subsets. Long-term non-adjustive ADV-conditioned mice showed increases in monocytes in the peripheral blood and of plasmacytoid dendritic cells (pDCs) and conventional dendritic cells (cDCs) in the spleen, while other myeloid subsets remained unchanged (Fig. S4A–C). In addition, the expression of inflammatory cytokines in cDCs and marginal zone (MZ) macrophages of the spleen remained unchanged (Fig. S4D), suggesting that the overall cytokine expression is increased in the spleen. These results demonstrate that long-term circadian misalignment causes a mild increase in myeloid cells in the spleen and blood. In conjunction with immune senescence, such as an increase in monocytes^36^, in the innate immunity^37^, our results of age-associated decline in adaptive immunity strongly support the acceleration of immune senescence and the induction of chronic inflammation by long-term circadian misalignment.

## Discussion

In this reverse-translational study to understand the pathophysiological mechanisms for adverse consequences of chronic circadian impairment, we examined the effects of long-term (up to 85 weeks from 12 weeks of age) CJL using adult wild-type mice subjected to non-adjustive (ADV) and adjustive (DEL) conditions. The mouse age span in this study may be analogous to adolescence until retirement in humans^38^.

Importantly, our study revealed that the lifespan of mice exposed to ADV conditioning was significantly shorter than that of mice exposed to control LD or DEL conditions (Fig. 1). In ADV-conditioned mice, in accordance with recent reports, we observed severe desynchronization of circadian oscillation within the SCN^20,21^, whereas we detected no apparent desynchronization of SCN neuronal rhythms in the SCN slices of LD-conditioned mice of similar age (103~112-week-old) (Fig. 2). These observations suggest that intra-SCN desynchronization may contribute to the initiation of circadian misalignment-associated pathophysiological mechanisms. Recently, light-dark cycle entrains peripheral tissues including liver without functional SCN clock^39,40^, therefore other pathways than SCN-dependent axis may also contribute to the deleterious consequences associated with the long-term perturbation of light-dark cycle. In any case, RNA-seq data of liver and kidney tissues obtained from 85-week ADV-conditioned mice indicated that the immune function and inflammation-related pathways were markedly activated (Fig. 3). Moreover, transcriptional factor (TF) enrichment analysis using the RNA-seq data revealed that the gene modules regulated by core clock components such as CLOCK, BMAL1, PER1, PER2, CRY1 and CRY2 were widely suppressed in ADV-conditioned liver (Fig. 4). These findings strongly suggest that the environmental perturbation by non-adjustive shifted condition of light-dark cycle resulted in the “reprogramming” of the circadian gene regulatory networks in the peripheral tissues. Moreover, some immune system-related transcriptional factors were revealed in the TF enrichment analysis (Fig. 4). Decreased expression of *Nfil3* may be related to the enrichment of Th17 in the intestine and the spleen^41^, consistent with the negative-regulation of NFIL3 activity in the ADV-conditioned mice compared with the LD-conditioned mice (Fig. 4). Furthermore, IRF8 activity appeared to be positively regulated in ADV-conditioned mice (Fig. 4). It has been reported that the increase in IRF8 promotes the increases in the myeloid cells including monocyte, DC, and pDC^42^. This is consistent with the elevated abundance of monocytes, pDCs, and cDCs in the peripheral blood or spleen of the ADV mice (Fig. S4A,C). These results demonstrate that immune network is markedly affected by the long-term non-adjustive CJL.

Recently, it has reported that age-related pathologies are associated with circadian clock^43,44^. Our results in this study suggest that the disruption of circadian rhythm itself play a critical role for acceleration of ageing processes in mice. A non-biased evaluation using comprehensive RNA-seq analysis to survey the physiological functions impaired by the circadian misalignment in the mouse liver revealed the activation of pathways associated with the immune system, in addition to suppression of metabolic pathways, as apparent pathophysiological consequences of exposure to ADV conditions for 85 weeks. Furthermore, we also observed the activation of immune system and immune disease-related pathways in 85-week ADV-conditioned kidneys (Fig. 3). Intriguingly, the subset analysis of immune cells in these ADV conditioned mice has shown the significant increases in SA-T, Tfh, Treg cells, and GC B cells (Fig. 6), which suggests that the acceleration of immune senescence and ageing-related changes in immune cell functions may play a critical role for establishing the pathophysiological consequence such as hepatic steatohepatitis.

Recent reports have indicated that immune system is broadly altered by the circadian dysregulation^45,46^. For example, circadian rhythms are closely linked with the sympathetic activity rhythm, and its dysregulation modulates immune functions^47–50^. In addition, endocrine dysregulation, often induced by jet-lag^2,26,27^, has been shown to be associated with immune senescence^51^. Moreover, since jet-lag induces dysbiosis in both mice and humans^52^, modification of gut microbiota may also contribute to the acceleration of immune senescence^53^. Together with these findings, our results suggest that the disruption of immune homeostasis induced by chronic circadian misalignment is a key mechanism underlying the systemic pathophysiological changes.

In addition to the enhancement of liver steatosis, we observed low-grade chronic inflammation with elevated fibrosis markers in the livers of ADV mice (Fig. 5). Recently, Kettner *et al*. reported that long-term CJL induced hepatocellular carcinogenesis in mice^48^. Although we failed to detect any macroscopically apparent hepatic tumors in our study, we observed common pathophysiological features — including chronic steatohepatitis — after long-term circadian misalignment. Since both immune senescence and low-grade chronic inflammation with elevated fibrosis markers are characteristic features of ageing^54^, these findings suggest that long-term circadian misalignment accelerates ageing via an accelerated immune senescence, ultimately aggravating chronic inflammation in mice (Fig. S5).

Chronic inflammation with immune senescence significantly increases risks of various chronic diseases such as metabolic syndrome, cardiovascular disease, inflammatory diseases, and cancers^55^. Our mouse model system of exposure to long-term non-adjustive shifted light conditions may mirror the pathophysiology of chronic circadian rhythm disruption in humans. In particular, our findings suggest that immune senescence and chronic low-grade inflammation are accelerated by long-term circadian perturbation via non-adjustive light-dark shift, leading to premature death. Therefore, immune homeostasis may be a crucial target for intervention against clock-associated diseases.

## Methods

### Animals and experimental conditions

Male C57BL/6J mice (10 weeks old) were purchased from SLC (Hamamatsu, Japan). The *Per2*^*Luc*^ knock-in mice were originally developed by Dr. Joseph Takahashi’s group^14^ and maintained in our facility. Mice were housed in plastic cages in light-shield mouse housing boxes (1800 × 360 × 520 mm) at a room temperature of 25.3 ± 0.3 °C with 55% humidity. The mice were acclimatized to the environment in the Animal Experimentation Center of the Kyoto Prefectural University of Medicine for 2 weeks. Mice were housed in groups (4–5 mice per cage) and light intensity was set at 200 lx within the cages. The mice were allowed ad libitum access to food and water, and were regularly subjected to observations to identify apparent abnormalities during captivity.

Chronic jet lag (CJL) conditions were designed based on our previous pilot study^22^. Mice acclimatized to the environment were divided into three groups of light-schedule conditions: LD-condition (light–dark condition with an 8:00–20:00 light period, n = 24); DEL-condition (8-h phase delay once every 7 days, n = 20); and ADV-condition (8-h phase advance once every 4 days, n = 20). Body weight of each mouse was measured once every two weeks during these CJL experimental periods.

For tissue sampling, blood was collected by cardiac puncture under deep terminal anesthesia by isoflurane and added to an equal volume of 0.5 mM EDTA/PBS. Then mice were decapitated and the livers or kidneys were collected and snap-frozen in liquid nitrogen for RNA-seq analysis or fixed in 10% neutral buffered formalin for histological analysis immediately. Mesenteric lymph nodes (mLNs), bone marrow from femora, spleens, and peripheral bloods were collected and used for flow cytometry analysis.

All experiments were approved by the Experimental Animals Committee, Kyoto Prefectural University of Medicine, and were performed in accordance with the institutional guidelines and Guidelines for Proper Conduct of Animal Experiments by the Science Council of Japan.

### Behavioral analysis

The behavioral analysis of the mice exposed to the CJL conditions was performed as our previous report^22^. Each mouse was housed separately in a cage (170 × 350 × 145 mm) with a 120-mm diameter running wheel (SANKO, Osaka, Japan). The wheel-running frequency was measured by counting the number of signals from a magnet sensor (59070–010, Littelfuse Inc., Chicago, IL, USA). ClockLab software (Actimetrics, Wilmette, IL, USA) was used to analyze and display the behavioral activity in wheel revolutions per 5-minute bin.

### SCN slice culture and bioluminescence recording

The SCN slice culture was carried out as previously reported^56^. Briefly, the 200-µm thickness of the SCN from male and female *Per2*^*Luc*^ knock-in mice was cultured in a recording medium [DMEM supplemented with 15 mM HEPES, 1.2 g/L NaHCO_3_, 20 mg/L kanamycin, 5 µg/mL insulin, 100 µg/mL human transferrin, 100 µM putrescine, 20 nM progesterone, and 30 nM sodium selenite (Sigma)] on a culture membrane (Millicell-CM; Millipore). The bioluminescence images were acquired every hour with a 59-min exposure duration by using an integrated incubator-microscope system (LV100 and LV200; Olympus). The analysis was performed using AquaCosmos 2.6.3.6 software (Hamamatsu Photonics) and ImageJ 1.47 v software.

### Histological analysis

The livers fixed in 10% neutral buffered formalin were embedded in paraffin and subjected to thin-slice section preparation, followed by haematoxylin and eosin (H&E) staining (New Histo. Science Laboratory Co., Tokyo, Japan). The number of cells in 0.1 mm^2^ was counted with a BZ-X710 microscope (KEYENCE).

### RNA-seq

Mouse livers and kidneys collected between 13 and 16 hours after the last light-on were homogenized in TRIzol reagent (Thermo Fisher Scientific) and total RNA was extracted using RNeasy column (QIAGEN) according to the manufacturer’s instructions. Poly(A)-enriched stranded RNA sequencing was carried out by Takara Bio, Japan, on Illumina HiSeq 2500 with 100-bp paired-end reads and by Macrogen Japan on Illumina NovaSeq 6000 with 101-bp paired-end reads. After adaptor sequences were trimmed using Trimmomatic^57^, the sequence reads were mapped to the mouse genome (GRCm38/mm10) using STAR^58^ as described previously^59^. To obtain reliable alignments, the reads with a mapping quality of less than 10 were removed by SAM tools^60^. The University of California, Santa Cruz (UCSC) known canonical gene set (32,989) were used for annotation, and the reads mapped to the exons were quantified using Homer^61^. We assumed that a gene was expressed if there were more than 0.5 reads per million reads mapped on average in the exon of the gene. Differential gene expression of RNA-seq was determined using DESeq2^62^. Enrichment analysis was based on KEGG functional hierarchy^63^. *P* values for the enrichment test were calculated by GAGE algorithm^64^, and the FDR was calculated from the *p* value for multiple testing with Benjamini-Hochberg procedure. The enrichment results were visualized using FuncTree^65^. Enrichment analysis to evaluate the effects of transcription factors on their binding target genes was performed as described previously^66^.

### Antibodies and flow cytometry

Before the tissue sampling, the mice were housed in LD condition for 5–8 days without the phase shift. The mLNs, spleen, bone marrow, and peripheral bloods were collected between 1 and 4 hours after the last light-on and the single cell suspension was prepared. Spleen and bone marrow cells were treated by ACK buffer to lyses red blood cells. After cell counting by Celltacα MEK6450 fully automatic blood cell counter (Nihon Kohden Corp.), peripheral blood cells were treated by ACK buffer.

Fluorescent dye- or biotin-conjugated antibodies were purchased from BD Biosciences, eBioscience, Biolegend, and TONBO: CD3ε (145-2C11), TCRβ (H57-597), CD4 (RM4.5), PD-1 (29F.1A12), CD44 (IM7), CXCR5 (L138D7), CD153 (RM153), CD8α (53-6.7), CD25 (PC61.5), NK1.1 (PK136), γδTCR (GL3), B220 (RA3-6B2), CD19 (MB19-1), IgM (RMM-1), CD93 (AA4.1), CD23 (B3B4), CD95 (15A7), GL7, IgG1 (RMG1-1), IgA (C10-3), CD11c (N418), Gr-1 (RB6-8C5), F4/80 (BM8), CD11b (M1/70), CD115 (AFS98), PDCA-1 (927), MHC-II (M5/114.15.2), c-kit (2B8), CD27 (LG.3A10), TER119, IFN-γ (XMG1.2), IL-4 (11B11), and IL-17A (TC11-18H10.1). Biotinylated monoclonal antibodies were detected with PE- or BV421-conjugated streptavidin (BD Pharmingen, BioLegend). Viable cells were analyzed with FACSVerse flow cytometers (BD Biosciences) with FlowJo software. Values in quadrants, the gated area, and interval gates indicate the percentages of each population in all figures.

### Cell culture

mLN cells were cultured in RPMI 1640 medium supplemented with 10% FBS, 10 mM HEPES (pH 7.4), 50 μM 2-mercaptoethanol, streptomycin, and penicillin. For detection of cytokine production, cells were stimulated with PMA (50 ng/ml, Cayman) and ionomycin (2 μg/ml, Cayman) for 3 hours in the presence of Brefeldin A (5 μg/ml, Cayman). After stimulation, the cells were fixed by IC Fixation Buffer (Invitrogen), permeabilized, and stained with anti-IFNγ, IL-4, and IL-17A antibodies.

### Real time RT-PCR

cDC (CD11c^high^) and MZ macrophages (CD11b^+^F4/80^low^CD11c^low^) in CD19^−^CD3^−^NK1.1^−^Gr-1^−^TER119^−^PDCA-1^−^ splenocytes were sorted by FACSAria II cell sorter (BD Bioscience) and suspended with Sepasol RNA I Super G (Nacalai Tesque). Total RNA was purified and treated with RNase-free DNase (Takara Bio). After DNase inactivation, total RNA was reverse-transcribed by using ReverTraAce (TOYOBO) and random primers (Invitrogen). cDNA was measured by real-time RT-PCR by using SYBR Green Master Mix (QIAGEN) in an ABI7500 real time PCR system (Applied Biosystems). RNA expression was normalized by *Gapdh* mRNA using cDNA from whole splenocytes of LD-conditioned mice. The following primer sets were used: *Gapdh*, 5′-CCTCGTCCCGTAGACAAAATG-3′ and 5′-TCTCCACTTTGCCACTGCAA-3′; *Tnf*, 5′-GTCAGCCGATTTGCTATCTC-3′ and 5′-ACAGAGCAATGACTCCAAAG-3′; *Il1b*, 5′-GTCCTGATGAGAGCATCCAG-3′ and 5′-TCATATGGGTCCGACAGCAC-3′; *Il6*, 5′-GTCTTCTGGAGTACCATAGC-3′ and 5′-TGGTCTTGGTCCTTAGCCAC-3′.

### Statistical analysis and data analysis

For the survival analysis, the Kaplan-Meier survival curves were constructed by R 3.4.3 software. The stratified Cox proportional hazard regression model analysis was performed by using SAS 9.4 software (SAS institute, Inc., Cary, NC, USA).

For fast Fourier transform (FFT) analysis, the raw data was detrended by subtracting a 24-h moving average and the relative spectral power density (relative power) at the peak within the range of 21–26 h was obtained by using Microsoft Excel 2010.

For calculating amplitudes of single-cell bioluminescence traces, the detrended data were used for the analysis. Cosine wave fitting was carried out with the following equation, using GraphPad Prism 6.0 software:

$$f(t)=A\,\cos (2\pi \frac{t}{T}-\varphi )$$

where *A*: amplitude, *T*: period, and *φ*: acrophase.

The statistical analyses mentioned in figure legends was performed by R 3.4.3, SAS 9.4, Microsoft Excel 2007 and 2010, and GraphPad Prism 6.0 software otherwise noted. The significance was defined as *P* < 0.05 unless otherwise stated.

## Supplementary information

Supplementary Information.

## Data Availability

RNA sequencing data are available at the Gene Expression Omnibus (GSE142248). All other datasets generated in this study are available from the corresponding author upon reasonable request.
