# Supplementary material for: Chronic circadian misalignment accelerates immune senescence and abbreviates lifespan in mice
Source: Sci Rep. 2020 Feb 13;10:2569. doi: 10.1038/s41598-020-59541-y (PMC7018741; doi:10.1038/s41598-020-59541-y)
Supplement: Supplementary file 1 — Supplementary Information. [file 41598_2020_59541_MOESM1_ESM.pdf]

Chronic circadian misalignment accelerates immune senescence and abbreviates lifespan in mice

Hitoshi Inokawa[1]†, Yasuhiro Umemura[1]†, Akihiro Shimba[2]†, Eiryo Kawakami[3,4], Nobuya Koike[1], Yoshiki Tsuchiya[1], Munehiro Ohashi[1], Yoichi Minami[1], Guangwei Cui[2], Takuma Asahi[2,8], Ryutaro Ono[1], Yuh Sasawaki[1], Eiichi Konishi[5], Seung-Hee Yoo[6], Zheng Chen[6], Satoshi Teramukai[7], Koichi Ikuta[2] and Kazuhiro Yagita[1]*

[1]Department of Physiology and Systems Bioscience, Kyoto Prefectural University of Medicine, Kyoto 602-8566, Japan

[2]Laboratory of Immune Regulation, Department of Virus Research, Institute for Frontier Life and Medical Sciences, Kyoto University, Kyoto 606-8507, Japan

[3]Medical Sciences Innovation Hub Program, RIKEN Center for Integrative Medical Sciences, Yokohama, Kanagawa 230-0045, Japan

[4]Artificial Intelligence Medicine, Graduate School of Medicine, Chiba University, Chiba 260-0856, Japan

[5]Department of Surgical Pathology, Kyoto Prefectural University of Medicine, Kyoto 602-8566, Japan

[6]Department of Biochemistry and Molecular Biology, The University of Texas Health Science Center at Houston, 6431 Fannin St., Houston, TX 77030

[7]Department of Biostatistics, Kyoto Prefectural University of Medicine, Kyoto 602-8566, Japan

[8]Graduate School of Medicine, Kyoto University, Kyoto 606-8501, Japan

*Correspondence to: Kazuhiro Yagita (kyagita@koto.kpu-m.ac.jp)

†These authors contributed equally to this work.

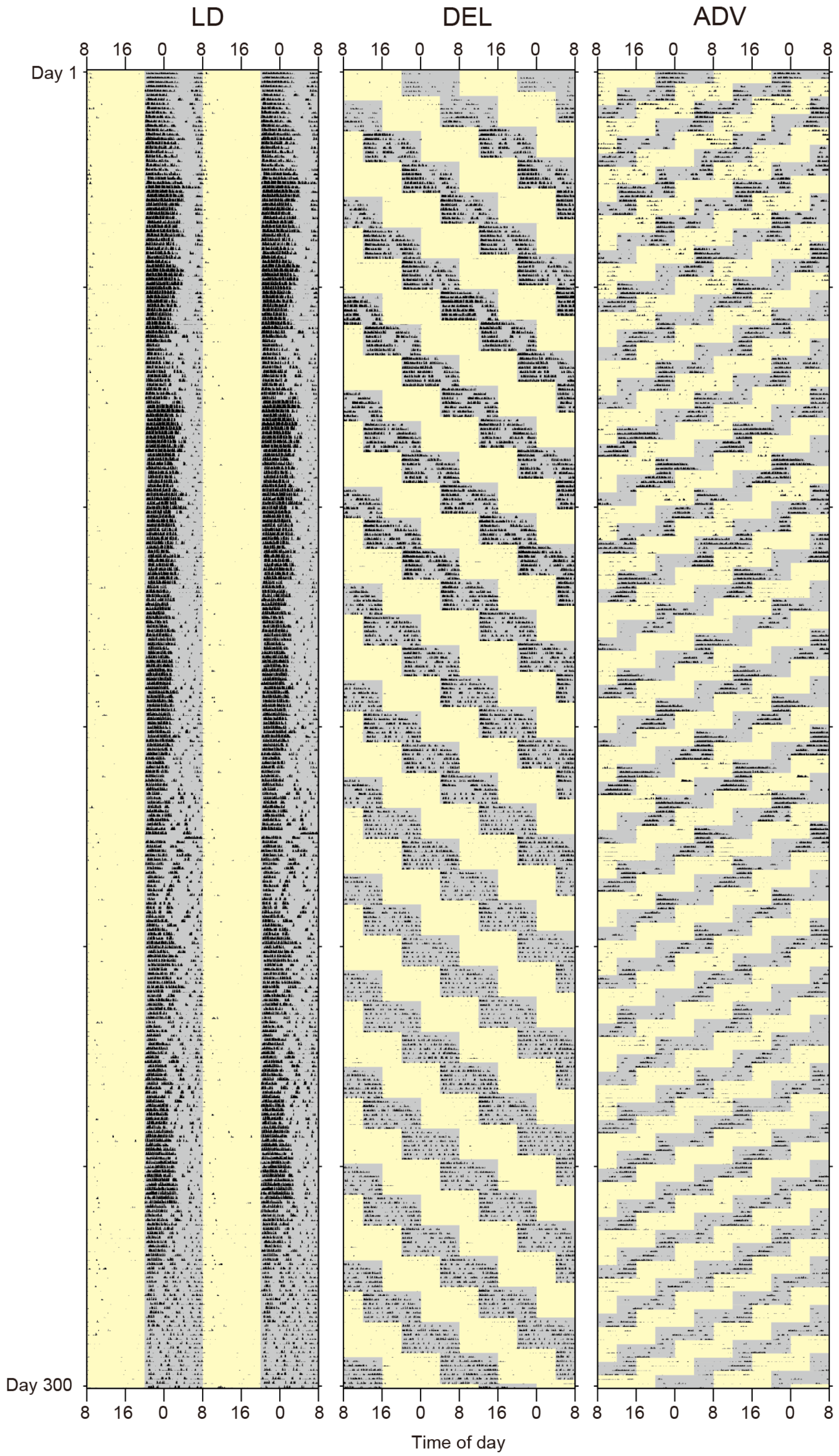

Figure S1. Wheel running rhythms. Representative double-plotted actograms of wheel running from mice kept under the LD-, DEL-, and ADV-conditions until 300 days after the beginning of the three light–dark cycle conditions. Yellow or grey shades indicate light or dark phases, respectively.

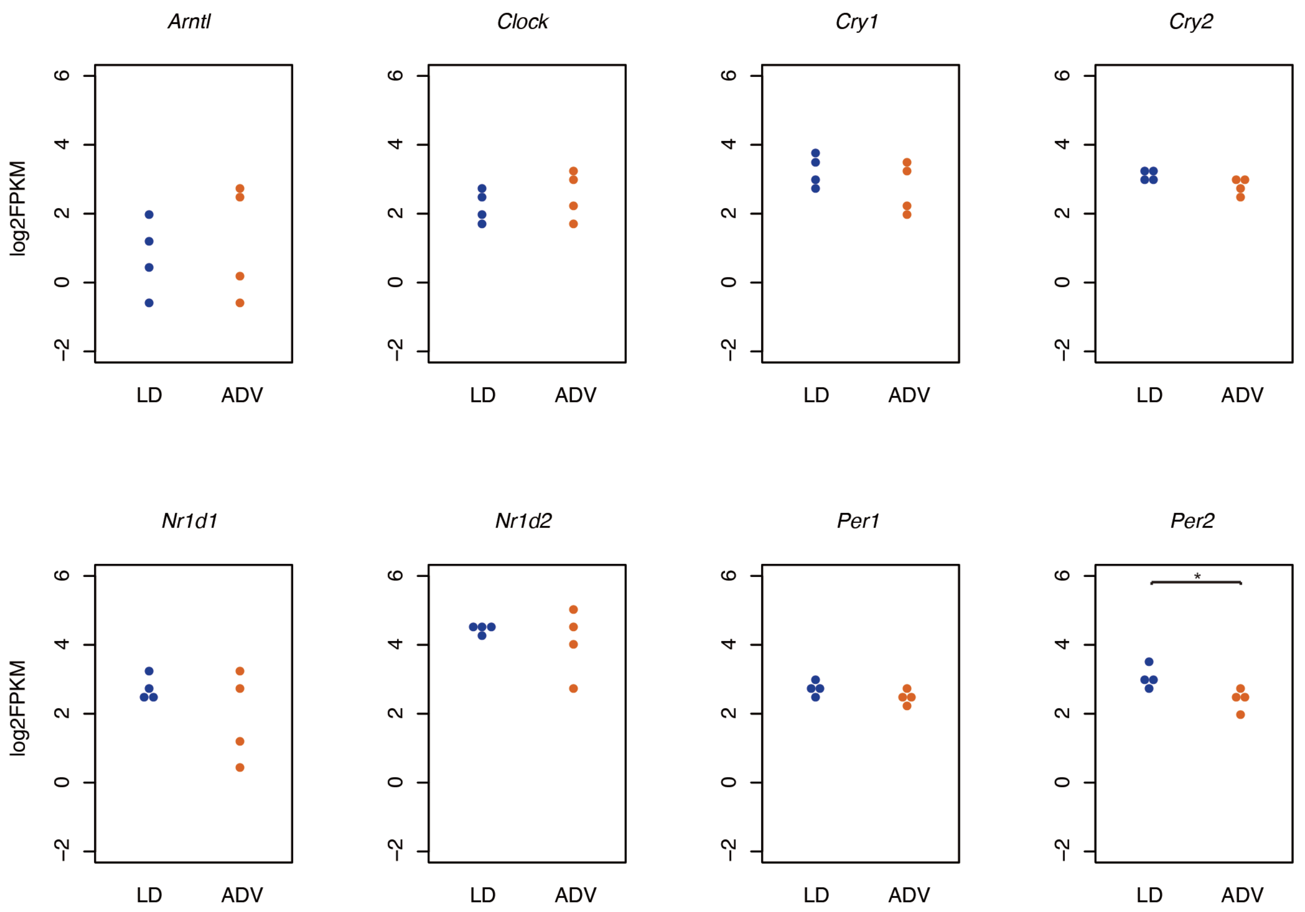

Figure S2. The core clock gene expressions in the 85-w CJL livers by RNA-seq. Asterisks indicate DESeq2 significance (FDR < 0.05).

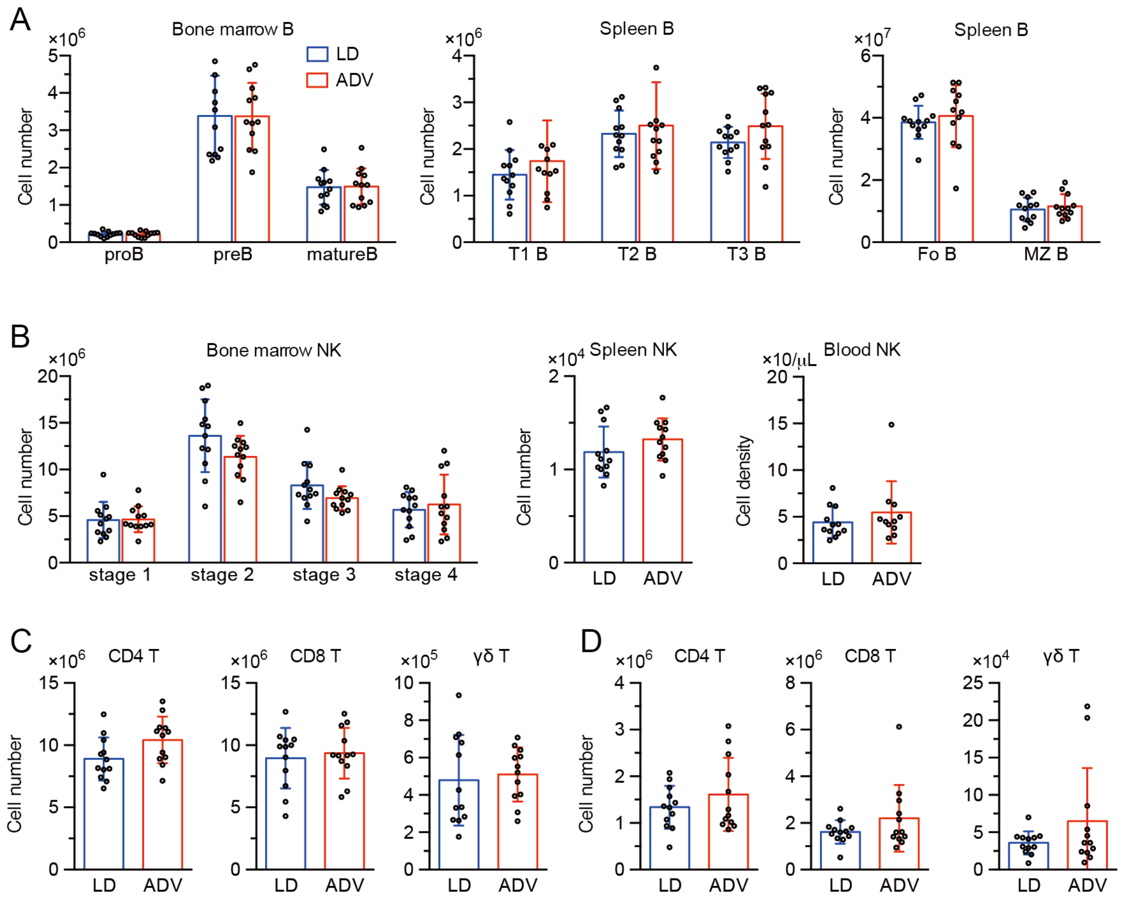

Figure S3. Long-term non-adjustive CJL conditions do not affect lymphocyte development. (A) Cell numbers of pro-B (CD19^{+}B220^{+}c-kit^{+}IgM^{-}), pre-B (CD19^{+}B220^{+}c-kit^{-}IgM^{-}), and mature B (CD19^{+}B220^{+}IgM^{+}) cells in bone marrow and transitional 1 (T1) B (CD19^{+}B220^{+}CD93^{+}CD23^{low}IgM^{high}), T2 B (CD19^{+}B220^{+}CD93^{+}CD23^{high}IgM^{high}), T3 B (CD19^{+}B220^{+}CD93^{+}CD23^{high}IgM^{low}), marginal zone B (MZ B, CD19^{+}B220^{+}CD93^{-}CD23^{high}IgM^{low}), and follicular B (Fo B, CD19^{+}B220^{+}CD93^{-}CD23^{low}IgM^{high}) cells in spleens of LD- and ADV-conditioned mice (n = 12). (B) Cell numbers of natural killer (NK) cells at stage 1 (CD3^{-}NK1.1^{+}CD27^{-}CD11b^{-}), stage 2 (CD3^{-}NK1.1^{+}CD27^{+}CD11b^{-}), stage 3 (CD3^{-}NK1.1^{+}CD27^{+}CD11b^{+}), and stage 4 (CD3^{-}NK1.1^{+}CD27^{-}CD11b^{+}) in bone marrow (n = 12) and NK cells (CD3^{-}NK1.1^{+}) in spleens (n = 12) and cell density of NK cells in peripheral blood (n = 11–12) of LD- and ADV-conditioned mice. (C) Cell numbers of CD4 T (TCRβ^{+}CD4^{+}CD25^{-}), CD8 T (TCRβ^{+}CD8^{+}), and γδ T (TCRβ^{-}γδTCR^{+}) cells in spleens of LD and ADV mice (n = 12). (D) Cell numbers of CD4 T (TCRβ^{+}CD4^{+}CD25^{-}), CD8 T (TCRβ^{+}CD8^{+}), and γδ T (TCRβ^{-}γδTCR^{+}) cells in mLNs of LD- and ADV-conditioned mice (n = 12). Data are means ± SD.

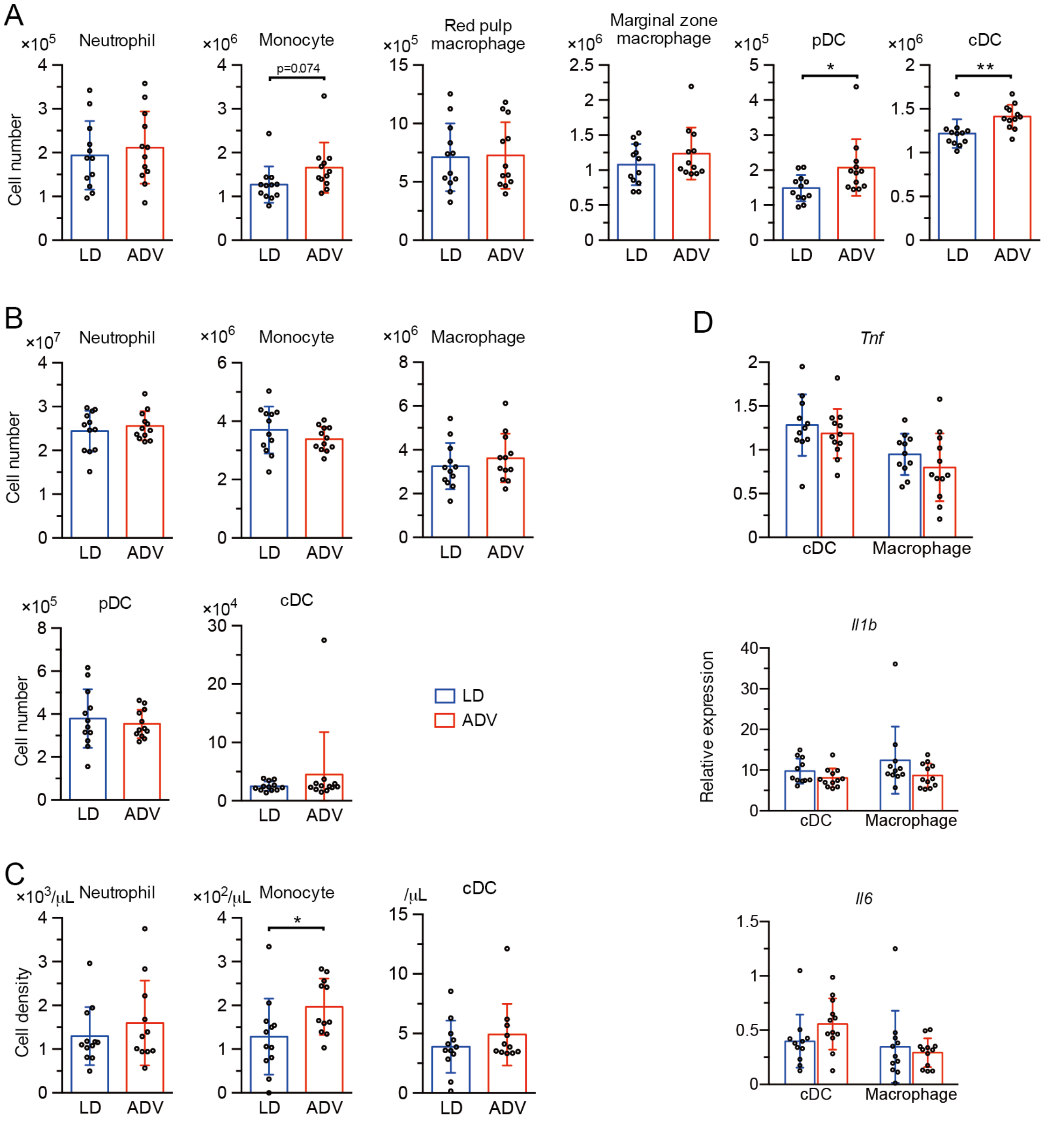

Figure S4. Long-term non-adjustive CJL conditions induce mild increases in monocytes and dendritic cells. (A) Cell numbers of neutrophils ($CD11b^+Gr$-$1^+CD3^-CD19^-$), monocytes ($CD115^+CD11b^+Gr$-$1^-CD3^-19^-$), red pulp macrophages ($CD11b^+F4/80^{low}CD3^-CD19^-CD115^-$), marginal zone macrophages ($CD11b^{low}F4/80^{high}CD3^-CD19^-CD115^-$), plasmacytoid dendritic cells (pDCs, PDCA-1^+MHCII^{high}), and conventional DCs (cDCs, $CD11c^+MHCII^{high}PDCA$-1^-) in spleens of LD- and ADV-conditioned mice (n = 12). (B) Cell numbers of neutrophils, monocytes, macrophages ($CD11b^+F4/80^{low}Gr$-$1^-CD3^-19^-115^-$), pDCs, and cDCs in bone marrow of LD- and ADV-conditioned mice (n = 12). (C) Cell density of neutrophil ($CD11b^+Gr$-1^+), monocyte ($CD115^+CD11b^+Gr$-1^-), and cDC ($CD11c^+MHCII^{high}$) in peripheral blood of LD- and ADV-conditioned mice (n = 11–12). (D) qRT-PCR analysis of *Tnf*, *Il1b*, and *Il6* mRNA of cDCs and marginal zone macrophages in spleens of LD- and ADV-conditioned mice (n = 11–12). Data are means ± SD. Two-tailed Student's *t*-test, **P* < 0.05, ***P* < 0.01, ****P* < 0.001.

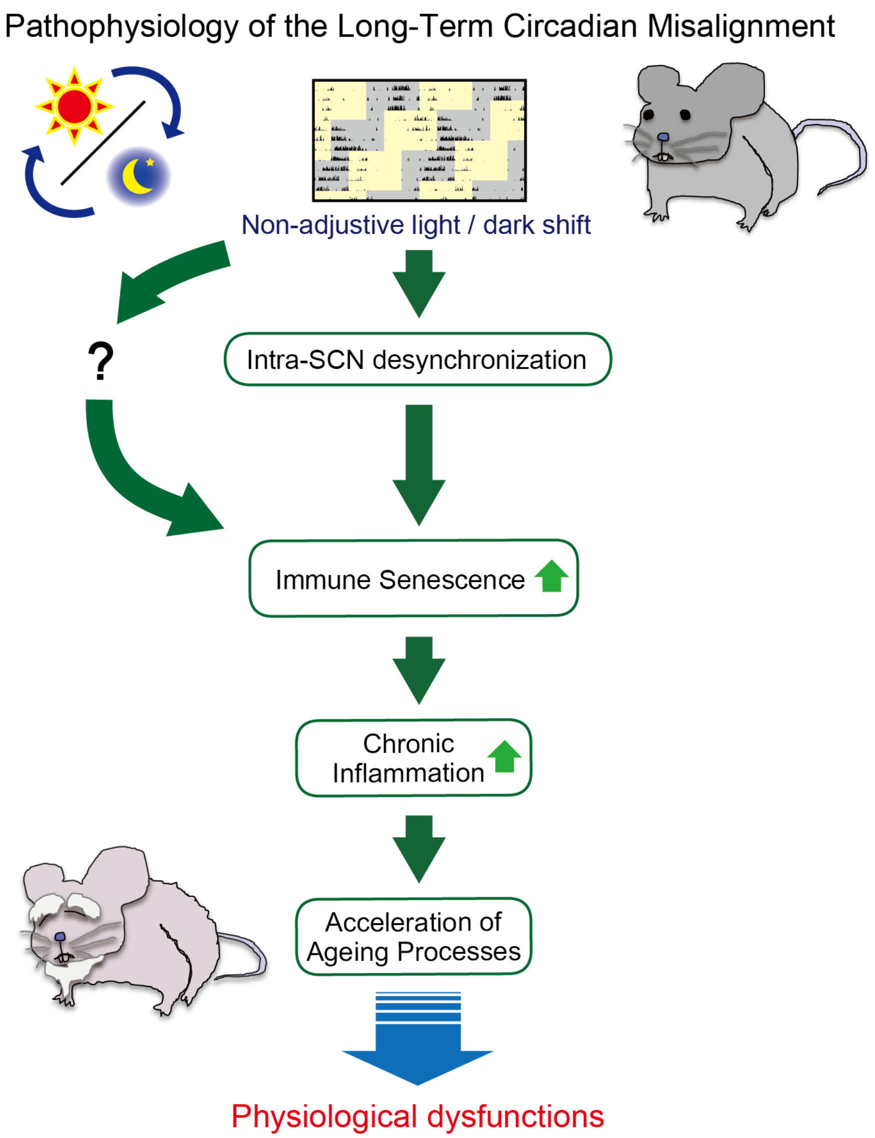

Figure S5. Schema for systemic ageing processes via 'SCN desynchronization–accelerated immune senescence–enhanced chronic inflammation' axis.
